# Preparation and structural analysis of fucomannogalactan and β-1,6-glucan from *Grifola frondosa* mycelium

**DOI:** 10.3389/fchem.2023.1227288

**Published:** 2023-08-07

**Authors:** Jie Geng, Guining Wang, Jiao Guo, Xiao Han, Yunhe Qu, Yifa Zhou, Guihua Tai, Lin Sun, Hairong Cheng

**Affiliations:** ^1^ Jilin Province Key Laboratory on Chemistry and Biology of Changbai Mountain Natural Drugs, Glycoconjugate Engineering Research Center of the Ministry of Education, School of Life Sciences, Northeast Normal University, Changchun, China; ^2^ Central Laboratory, Changchun Normal University, Changchun, China

**Keywords:** *Grifola frondosa*, mycelium, fucomannogalactan, glucan, structure analysis

## Abstract

**Introduction:** Polysaccharides, key components present in *Grifola frondosa*, can be divided into those derived from fruiting bodies, mycelium, and fermentation broth based on their source. The structure of *G. frondosa* fruiting body-derived polysaccharides has been fully characterized. However, the structure of *G. frondosa* mycelium-derived polysaccharides remains to be elucidated.

**Methods:** In this study, we obtained mycelia from *G. frondosa* by liquid fermentation and extracted them with water and alkaline solution. Then, the mycelia were isolated and purified to obtain homogeneity and systematically characterized by methylation and FT infrared (FT-IR) and nuclear magnetic resonance (NMR) spectroscopy.

**Results and discussion:** Structural analysis showed that two neutral fractions (WGFP-N-a and AGFP-N-a_1_) have a common backbone composed of α-1,6-D-Me-Gal*p* and α-1,6-D-Gal*p* that were substituted at O-2 by 1,2-Man*p*, α-1,3-L-Fuc*p*, and α-T-D-Man*p* and thus are identified as fucomannogalactans. WGFP-A-a, AGFP-A-b, and AGFP-A-c are β-1,6-glucans with different molecular weights and are branched with β-1,3-D-Glc*p* and T-D-Glc*p* at the O-3 of Glc. Our results provide important structural information about *G. frondosa* mycelium-derived polysaccharides and provide the basis for their further development and application.

## 1 Introduction

*Grifola frondosa* belongs to the subphylum Basidiomycota, class Laminaria, order Aphyllum, family Polyporaceae, and genus Trichomycota. *Grifola frondosa* is an edible and medicinal mushroom, with significant developmental and research value. It is very popular in China, Japan, and other Asian countries because of its flavor and nutritional value. *Grifola frondosa* has many active ingredients, such as polysaccharides (especially β-1,6-glucans and β-1,3-glucans), glycoproteins, ergosterol, polyphenol yellow, and fungal SOD enzyme (Švagelj et al., 2008; Wu et al., 2021). These polysaccharides can be divided into those derived from fruiting bodies, mycelium, and fermentation broth based on their source (Santos Arteiro et al., 2012). The structure of *G. frondosa* fruiting body-derived polysaccharides has been fully characterized. However, the structure of *G. frondosa* mycelium-derived polysaccharides remains to be elucidated. Recently, polysaccharides from *G. frondosa* mycelium have received considerable attention due to their anti-tumor, immune regulatory, antioxidant, and other properties (Zhuang et al., 1994; Nie et al., 2006; Cui et al., 2007; Zhao et al., 2016; Wu et al., 2021).

Given that *G. frondosa* mycelium-derived polysaccharides display numerous pharmacological activities, analyzing their structures is crucial for their development and utilization. Available reports have provided limited information about the structural characteristics of *G. frondosa* mycelium-derived polysaccharides. Iino et al. (1985) were the first to extract the Grifolan component from *G. frondosa* mycelium under cold alkaline conditions. Grifolan is a β-1,3-glucan with 1,6-Glc*p* branches. Adachi et al. (1994) extracted a purified fungal β-1,3-D-glucan with 0.5% citrate buffer from *G. frondosa* mycelium. An α-configuration proteoglycan GFPS1b with a molecular weight of 21 kDa was obtained from *G. frondosa* mycelium and found to be composed of Glc, Gal, and arabinose (Ara) at a molar ratio of 4:2:1. GFPS1b has a backbone consisting of α-1,4-Gal*p* and α-1,3-Glc*p* (Cui et al., 2007). Zhao et al. (2016) extracted the mycelium of *G. frondosa* with hot water at 80°C to obtain a polysaccharide GRP1 with a molecular weight of 40.5 kDa. Moreover, during the process of liquid fermentation, many factors, such as the source of bacteria, fermentation duration, medium selection, and polysaccharide extraction method, may impact the structure of the polysaccharide.

To fully characterize polysaccharides from *G. frondosa* mycelium, we prepared and investigated a series of water-soluble and alkali-soluble polysaccharides. Our results will enhance the structural information about *G. frondosa* mycelium-derived polysaccharides and provide direction for the development and application of *G*. *frondosa* mycelium-derived polysaccharides as functional food.

## 2 Materials and methods

### 2.1 Materials

The *Grifola frondosa* CTS8 strain was purchased from BNCC (Henan, China). DEAE-cellulose was purchased from Shanghai Chemical Reagent Research Institute (Shanghai, China). Sepharose CL-6B was purchased from GE healthcare (Pittsburgh, United States). Bio-Gel P-2 was purchased from Bio-Rad (California, United States). β-1,3-Glucanase and α-glucoamylase were purchased from Megazyme (Ireland). All other chemicals used were of analytical grade and commercially available or produced in China.

### 2.2 Liquid fermentation culture

Strain activation and preservation: The prepared PDA culture was sterilized at 115°C for 30 min. After cooling, the slanted strains of *G. frondosa* were kept under aseptic conditions, and two pieces (∼0.5 cm × 0.5 cm) were cut. The blocks were inoculated on the PDA plate medium, placed in an incubator maintained at a constant temperature of 25°C for 14 days, and transferred to 4°C for storage after the medium surface was completely covered by mycelia.

For liquid seed culture, the prepared medium was dispensed into 2-L conical flasks which were filled with 1 L of liquid. The flasks were sterilized at 115°C for 30 min, and then each flask was inoculated under aseptic conditions. Each Erlenmeyer flask was connected to 25 activated *G. frondosa* flat plates (∼1 cm × 1 cm) and placed them in a shaker maintained at a constant temperature of 25°C. The flasks were cultured at 160 rpm for 7 days to obtain a large number of uniform-sized mycelium balls.

The fermentation culture was carried out in a 5-L fermenter containing 3 L of the sterilized fermentation medium, which had been prepared and cooled under aseptic conditions (115°C for 30 min). The *G. frondosa* seed solution was inoculated with 10% of the inoculum and cultivated for 6 days while being fed in batches. Real-time monitoring for the pH value and dissolved oxygen value was conducted, and ventilation volume and stirring speed were controlled.

### 2.3 Polysaccharide extraction and purification

After drying, the mycelium was extracted with distilled water (material/dH_2_O, w/v, 1:20) twice at 100°C for 3 h. Extracts were concentrated under vacuum at 60°C and precipitated using four volumes of 95% ethanol at 4°C for 12 h. After centrifugation (4,000 rpm, 15 min), the precipitate was collected, redissolved in dH_2_O, frozen at −80°C for 30 min, and freeze-dried using an Alpha 2-4 LD plus freeze dryer (Christ, Germany). A water-soluble polysaccharide (WGFP) was obtained, and mycelium residues after water extraction were obtained with 0.5 M of NaOH solution (material/alkali, w/v, 1:25) three times at 80°C for 3 h. After neutralization, the extract was concentrated *in vacuo* at 60°C and precipitated at 4°C using four volumes of 95% ethanol for 12 h. After centrifugation (4,000 rpm, 15 min), the precipitate was collected and re-dissolved in water, followed by dialysis and lyophilization. The alkali-soluble polysaccharide AGFP was obtained.

WGFP and AGFP were dissolved in distilled water, applied on a DEAE-cellulose column (8.0 × 20 cm, Cl^−^), and eluted with distilled water and 0.3 M NaCl, yielding a neutral polysaccharide and an acidic polysaccharide fraction, respectively. All fractions were further purified using a Sepharose CL-6B column, resulting in homogeneous neutral and acidic polysaccharide fractions.

### 2.4 General methods

The total carbohydrate content was determined by the phenol–sulfuric acid protocol with glucose as the standard (DuBois et al., 1956). The uronic acid content was determined by using the colorimetric method proposed by Filisetti-Cozzi and Carpita (1991) with glucuronic acid as the standard. The protein content was determined by using the Bradford assay with bovine serum albumin (BSA) as the standard (Sedmak Jj Fau - Grossberg and Grossberg, 1977). The ash content was determined by using the muffle furnace burning method. A glycogen-like polysaccharide was detected by the I_2_–KI assay (Wang et al., 2010). Molecular weights were determined by high-performance gel-permeation chromatography (HPGPC). HPGPC was carried out at 40°C using a TSK-gel G-3000PW_XL_ column (7.8 × 300 mm, Tosoh, Japan) connected to a Shimadzu high-performance liquid chromatography (HPLC) system. The column was pre-calibrated with dextrans as the standard. Polysaccharide samples (5 mg/mL) were dissolved in 0.2 M NaCl at a flow rate of 0.6 mL/min and monitored using a refractive index RID-10A detector (Shimadzu, Tokyo, Japan).

### 2.5 Monosaccharide composition analysis

Monosaccharide composition was analyzed by using an external standard method. According to the retention time of nine monosaccharide standards, the monosaccharide composition of polysaccharide samples was determined. The content of each monosaccharide was determined based on the peak area.

A polysaccharide sample (2 mg) was hydrolyzed first with anhydrous methanol containing 1 M HCl at 80°C for 16 h and then with 2 M TFA at 120°C for 1 h. Hydrolyzed monosaccharides and nine monosaccharide standards were derived using 1-phenyl-3-methyl-5-pyrazolone (PMP) and analyzed by HPLC as previously described (Wang et al., 2023). The column temperature was 35°C, and the detection wavelength was 245 nm. The flow rate was 1.0 mL/min, and the injection volume was 10.0 μL. The mobile phase was 80.8% PBS (0.1 M, pH 7.0) and 19.2% acetonitrile (v/v), and the detector used was an SPD-20A ultraviolet detection system.

### 2.6 Fourier transform infrared spectroscopy

The polysaccharide sample was ground with spectroscopic grade KBr and then pressed into a powder to form a pellet. Fourier transform infrared spectroscopy (FT-IR) spectra were obtained on the PerkinElmer Spectrum Two FT-IR spectrometer (PerkinElmer, United States) in the wavenumber range of 4,000–400 cm^−1^.

### 2.7 Methylation analysis

Methylation analysis was carried out according to the method proposed by Needs and Selvendran (1993). In brief, the polysaccharide sample (10 mg) was dissolved in DMSO (1.5 mL) and methylated with a suspension of NaOH/DMSO (1.5 mL) and iodomethane (2.0 mL). The reaction mixture was extracted with dichloromethane (CH_2_Cl_2_), and then the solvent was removed by vacuum evaporation. Complete methylation was confirmed by the disappearance of the -OH band (3,200–3,400 cm^−1^) in the FT-IR spectrum. The per-*O*-methylated polysaccharide was hydrolyzed subsequently using HCOOH (85%, 1 mL) for 4 h at 100°C and then with CF_3_COOH (2 M, 1 mL) for 6 h at 100°C. The partially methylated sugars in the hydrolyzate were reduced by NaBH_4_ and then acetylated. The resulting alditol acetates were analyzed by GC-MS (7890B-5977B, Agilent, United States) using an HP-5ms capillary column (30 m × 0.32 mm × 0.25 mm). The oven temperature was programed from 120°C (hold for 1 min) to 210°C (hold for 2 min) at a rate of 3 ^°^C/min and then up to 260°C (hold for 4 min) at a rate of 10 ^°^C/min. The temperature of both the inlet and the detector was set at 300°C. Helium was used as a carrier gas. The mass scan range was 50–500 m/z.

### 2.8 Congo Red experiment

Congo Red binds to polysaccharides that have a three-helix structure, thus exhibiting a characteristic redshift (Liu et al., 2011). Various concentrations of NaOH solutions were prepared: 0, 0.1, 0.2, 0.4, 0.6, and 0.8 M. Congo Red was dissolved in a solution of 80 μM. Polysaccharide samples were prepared at a concentration of 2 mg/mL, and 2.5 mL of 80 μM Congo Red was added to this solution followed by the addition of distilled water. Finally, 3 mL NaOH solution of various concentrations was added to make the final concentration 0–0.4 M. The solution was equilibrated at room temperature for 1 h and analyzed using an UV spectrophotometer covering the wavelength range of 400–700 nm to identify the maximum absorption wavelength.

### 2.9 NMR spectroscopy

^1^H and ^13^C NMR spectra were recorded at 20°C on a Bruker Avance 600 MHz spectrometer (Germany) with a Bruker 5 mm broadband probe, operating at 600 MHz for ^1^H NMR and 150 MHz for ^13^C NMR. Samples (20 mg) were dissolved in D_2_O (0.5 mL) and centrifuged to remove any undissolved substances. Data were analyzed using the standard Bruker software.

### 2.10 Enzymatic hydrolysis analysis

A measure of 20 mg AGFP-N-b was added with 4 mL dH_2_O to obtain a reaction substrate with a concentration of 5 mg/mL. When the sample was fully dissolved, saccharification enzyme was added at a rate of 1 U and allowed for a reaction period of 24 h at 37°C. At the end of 100°C for 10 min, the reaction mixture was centrifuged at 12,000 rpm for 10 min. The supernatant was collected after concentration and freeze-dried. Finally, the lyophilized sample was applied on the Bio-Gel P-2 gel column (1.6 × 100 cm). The eluate was ddH_2_O, and the elution flow rate was 0.15 mL/min. The elution peaks were detected by using the phenol-sulfuric acid method, the two main elution peaks were collected and freeze-dried. Then, the monosaccharide composition and methylation analysis were performed.

## 3 Results

### 3.1 Liquid fermentation culture

Maitake mycelium showed a dense white hyphae morphology on the solid-activated medium, as shown in Supplementary Figure S1A. After inoculation in a triangular vial for approximately 3 days, fine uniform bacterial bulbs developed, and in approximately 7 days hyphal bulbs were visibly denser (∼2 mm in diameter), and the bacterial liquid became acidic, as shown in Supplementary Figure S1B. The seed solution was inoculated in a 5-L fermentation tank (3 L of liquid loading) according to the inoculation amount of 10% (v/v), and sterility was maintained. The bacterial growth process can generally be seen in the stagnation period, linear growth period, stabilization period, and senescence period. The growth of mycelium was slow in approximately 0–2 days during the early stage of fermentation; the growth rate of mycelium was fast in 3–4 days; then, the mycelium biomass increased rapidly, while the pH decreased. The growth was slow, yet stable, in 5–6 days. The fermentation liquid gradually became viscous, and the fermentation was stopped. The mycelium morphology of the fermentation tank culture is shown in Supplementary Figure S1C.

The liquid fermentation culture product of the fermenter was collected, and the dried *G. frondosa* mycelium was obtained by centrifugation, washing, and drying. A single liquid fermentation process took 13 days, and 22.8 g of the dried mycelium was obtained per 3 L of culture. The mycelium biomass yield was 7.6 g/L.

### 3.2 Extraction of polysaccharides

The total polysaccharide content from *G. frondosa* mycelium termed WGFP (10.2 g) was obtained by boiling water extraction of 200 g dried *G. frondosa* mycelium. The extraction yield was 5.1%. Then, the residue was extracted by alkali solution to obtain the polysaccharide (AGFP). The yield was 19.4%.

The components of WGFP and AGFP were determined, and the results are shown in Supplementary Table S1. The sugar content (40.0%–50.0%) of WGFP and AGFP was similar, and they both contained low amounts of uronic acid (<5.0%) and high protein content (∼20.0%). WGFP also contained high ash (33.0%). After deproteinization of WGFP and AGFP, the protein content of WGFP decreased from 18.9% to 3.2%, and the protein content of AGFP decreased from 28.8% to 4.1%.

After complete acid hydrolysis and derivatization, HPLC was used to determine monosaccharide composition. The results are shown in Table 1. The monosaccharide molar ratio of WGFP was Glc:Man:Me-Gal:Fuc:Gal:GlcA:Xyl = 51.6:22.6:10.2:7.1:4.2:2.6:1.7. WGFP mainly contains Glc, Man, and Me-Gal. The monosaccharide molar ratio of AGFP was Glc:Man:Me-Gal:Fuc:Gal:GlcA:Xyl = 65.9:9.6:8.3:7.5:4.0:2.6:2.1. AGFP is mainly composed of Glc, with a small amount of Man, Me-Gal, and Fuc.

**TABLE 1:** Yield, molecular weight, I_2_-KI chromogenicity, and monosaccharide composition of WGFP, AGFP, WGFP-N-a, WGFP-A-a, AGFP-N-a, AGFP-A-b, AGFP-A-c, AGFP-N-a, AGFP-N-a_1_, and AGFP-N-a_2_.

Fraction	Yield (%)	Mw (kDa)	I_2_-KI	Monosaccharide composition (mol%)						
				Glc	Gal	Man	Fuc	Me-Gal	GlcA	Xyl
WGFP	5.1	—	-	51.6	4.2	22.6	7.1	10.2	2.6	1.7
AGFP	19.4	—	**+**	65.9	4.0	9.6	7.5	8.3	2.6	2.1
WGFP-N-a	52.5^a^	28.9	-	4.6	9.3	33.6	26.8	25.7	—	—
WGFP-A-a	73.2^b^	5.0	-	80.2	1.7	11.8	—	—	6.3	—
AGFP-N-a	75.5^a^	28.6	**+**	27.6	4.3	24.3	18.5	22.3	—	3.0
AGFP-A-b	14.4^b^	19.9	-	91.4	—	2.7	1.3	1.4	3.2	—
AGFP-A-c	63.3^b^	4.1	-	91.6	—	4.2	—	—	4.2	—
AGFP-N-a_1_	—	28.7	-	4.1	4.3	33.3	24.6	30.2	—	3.5
AGFP-N-a_2_	—	—	**+**	100.0	—	—	—	—	—	—

^a^ Yield represents the yield of the neutral polysaccharide homogeneous fraction relative to the neutral polysaccharide loading.

^b^Yield represents the yield of the acidic polysaccharide homogeneous fraction relative to the acidic polysaccharide loading.

### 3.3 Fractionation of polysaccharides

**FIGURE 1:**
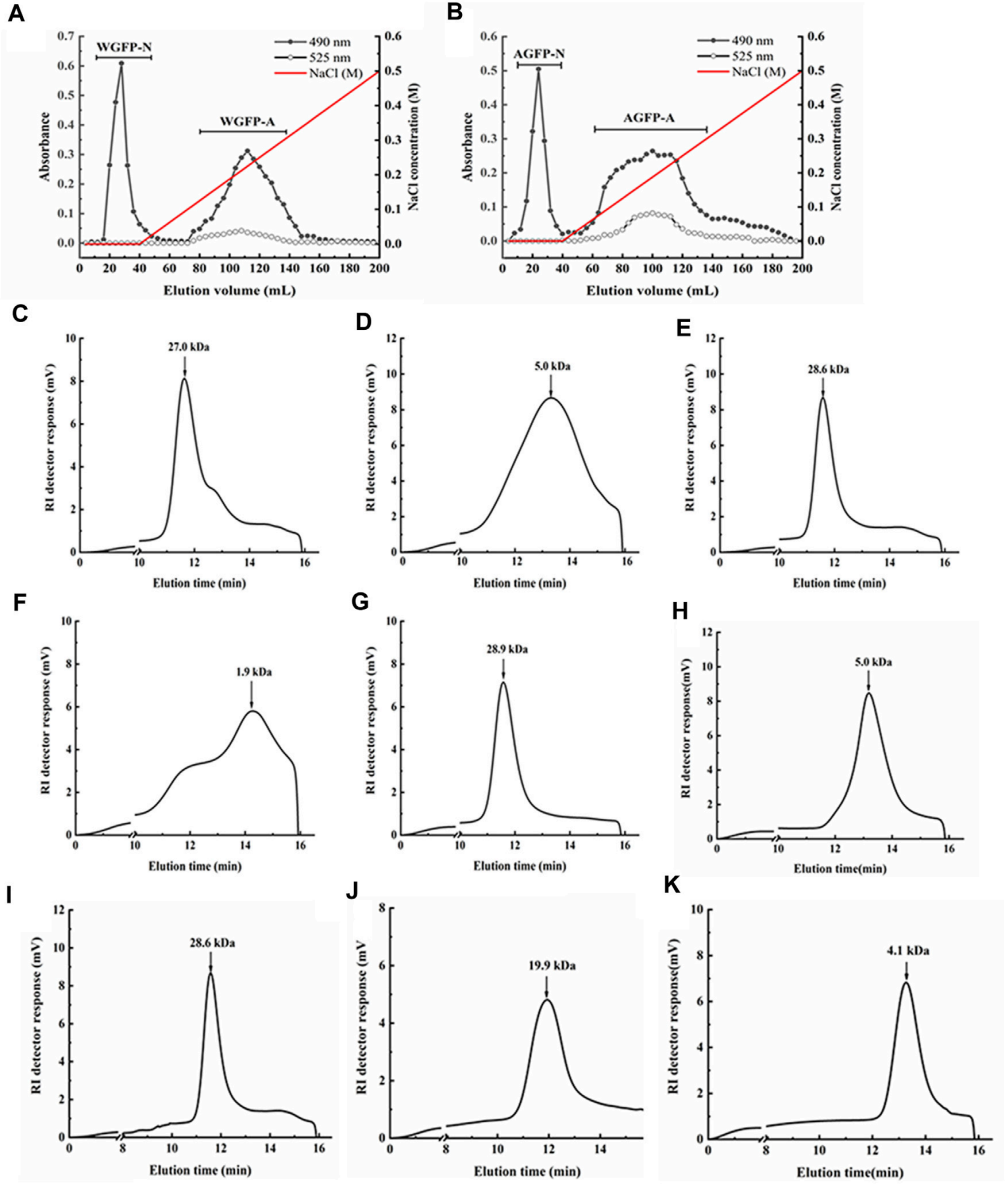
Elution profiles of **(A)** WGFP and **(B)** AGFP on the DEAE-cellulose column, eluted by a 547 linear gradient of NaCl, respectively (-●-, total sugar; -○-, uronic acid). The elution profiles of the polysaccharide fractions on HPGPC: **(C)** WGFP-N, **(D)** WGFP-A, **(E)** AGFP-N, **(F)** AGFP-A, **(G)** WGFP-N-a, **(H)** WGFP-A-a, **(I)** AGFP-N-a, **(J)** AGFP-A-b, and **(K)** AGFP-A-c.

WGFP and AGFP were both initially fractionated by anion-exchange chromatography. The neutral fraction was eluted with distilled water, and the acidic fraction was eluted with 0.3 M NaCl. These fractions were further separated by gel-permeation chromatography (Figure 1). Following separation, two purified neutral polysaccharide fractions (WGFP-N and AGFP-N) and two purified acid polysaccharide fractions (WGFP-A and AGFP-A) were obtained from different polysaccharides (Figure 1).

The weight-averaged molecular weights of WGFP-N, WGFP-A, AGFP-N, and AGFP-A were determined by HPGPC (Figure 1). The molecular weights of the two neutral polysaccharides, WGFP-N and AGFP-N, were 27 and 28.6 kDa, respectively. The molecular weights of the two acidic polysaccharides were low. The weight-averaged molecular weight of WGFP-A was ∼5 kDa, and the distribution range was wide. The molecular weight of AGFP-A was ∼1.9 kDa; however, the distribution range was not homogeneous. The Sephadex G-75 gel column was used for WGFP-N, AGFP-N, and AGFP-A to prepare polysaccharide fractions with homogeneous molecular weights. A Sephadex G-50 gel column was used for WGFP-A to prepare a polysaccharide fraction with a homogeneous molecular weight. This resulted in the preparation of five homogeneous polysaccharide fractions, WGFP-N-a, WGFP-A-a, AGFP-N-a, AGFP-A-b, and AGFP-A-c.

Monosaccharide compositions were determined by HPLC as shown in Table 1. The yields of WGFP-N-a and AGFP-N-a were 52.5% and 75.5%, with molecular weights of 28.9 and 28.6 kDa (Figure 1), respectively. WGFP-N-a and AGFP-N-a both mainly contained Man, Me-Gal, and Fuc, whereas AGFP-N-a also contained a small amount of Glc. The yields of WGFP-A-a, AGFP-A-b, and AGFP-A-c were 73.2%, 14.4%, and 63.3%, respectively, and their molecular weights were 5, 19.9, and 4.1 kDa (Figure 1), respectively. Monosaccharide composition showed that all fractions were mainly composed of Glc.

In addition to the results from the iodine chromogenic assay, saccharification enzyme was used to enzymatically hydrolyze AGFP-N-a to remove glucans. After enzymolysis, the sample was separated on a Bio-Gel P-2 gel column. The elution curve is shown in Supplementary Figure S3. Elution peaks were collected, and the initial elution peaks were named AGFP-N-a_1_ and AGFP-N-a_2_. The results of the monosaccharide composition analysis for AGFP-N-a, AGFP-N-a_1_, and AGFP-N-a_2_ are shown in Table 1. The content of Glc decreased from 27.6% to 4.1%, following enzymatic hydrolysis. The molecular weight distributions of AGFP-N-a and AGFP-N-a_1_ are shown in Supplementary Figure S4. The molecular weight distribution did not change after enzymolysis. Therefore, we assume that 27.6% of Glc was present as galactan.

### 3.4 FT-IR spectra analysis

**FIGURE 2:**
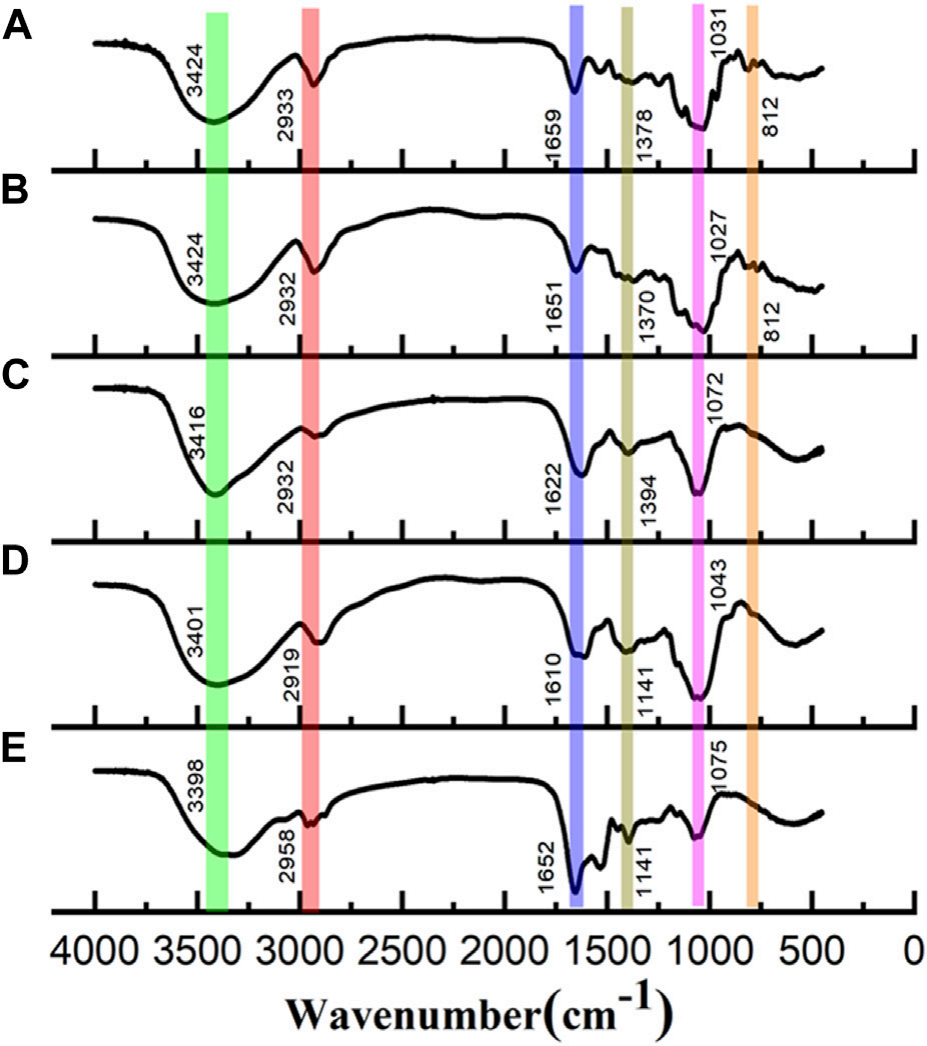
FT-IR spectra of **(A)** WGFP-N-a, **(B)** AGFP-N-a_1_, **(C)** WGFP-A-a, **(D)** AGFP-A-b, and **(E)** AGFP-A-c.

FT-IR spectra were used to characterize the primary functional groups in these polysaccharides and are shown in Figure 2. All polysaccharides showed the characteristic bands of carbohydrate compounds. The intense absorption band near 3,400 cm^−1^ (3,394 cm^−1^ or 3,374 cm^−1^) is associated with the stretching vibration of O-H, a characteristic of polysaccharides. The weak band observed at approximately 2,930 cm^−1^ (2,929 cm^−1^ or 2,927 cm^−1^) was attributed to C–H stretching of CH_2_ groups (Li and Shah, 2014). In addition, there was no significant absorption peak at 1730 cm^−1^, suggesting that all the polysaccharide fractions did not contain uronic acid (Zhang et al., 2023). Bands observed at 1,143 cm^−1^ are typical of C-O-C stretching vibrations (Zhang et al., 2015). The asymmetrical stretching bands observed at approximately 1,650 cm^−1^ (1,648 cm^−1^ or 1,645 cm^−1^) and the weaker symmetric stretching bands at approximately 1,409 cm^−1^ are attributed to asymmetric and symmetric stretching of C=O, respectively. The band observed at approximately 1,080 cm^−1^ (1,082 cm^−1^ or 1,075 cm^−1^) is the characteristic stretching vibration peak of pyran ring C-O-C, indicating the presence of the pyranose ring (Yan et al., 2019). Weak bands observed at approximately 858 cm^-1^ and 896 cm^-1^ indicate the presence of α-linked and β-linked glycosyl residues, respectively (Ning et al., 2021).

### 3.5 Structural analysis of polysaccharides

#### 3.5.1 Methylation analysis

The total ion chromatogram and fragment ion peaks from methylation are shown in Supplementary Figures S7, S8, and the results of glycosidic bond types and ratios are shown in Table 2. The structures of WGFP-N-a and AGFP-N-a_1_ are rather similar. Me-Gal and Gal exist in the form of 1,6-Gal and 1,2,6-Gal, respectively. Man residues mainly exist in the form of T-Man, and a small amount of 1,2-Man was also detected. Moreover, there was some 1,3-Fuc observed in the fraction. Based on this, we propose that both WGFP-N-a and AGFP-N-a_1_ are mainly composed of 1,6-Me-Gal*p* with a small amount of 1,6-Gal*p* to form the main chain, with some substitutions at O-2. T-Man*p*, 1,3-Fuc*p*, and 1,2-Man*p* are present as side chains. The branching degrees of WGFP-N-a and AGFP-N-a_1_ are 66.6% and 66.2%, respectively.

**TABLE 2:** Types and ratios of glycosidic bonds of WGFP-N-a and AGFP-N-a_1_.

Methylated sugar	Linkage	Molar ratio (%)		Mass fragment (m/z)
		WGFP-N-a	AGFP-N-a_1_	
2,3,4-Me_3_-Fuc*p*	T-	2.2	2.7	72,89,101,117,131,161,175
2,4-Me_2_-Fuc*p*	1,3-	20.1	23.1	89,101,117,131,159,173,233
2,3,4-Me_3_-Gal*p*	1,6-	13.1	13.8	87,101,117,129,161,173,189,233
3,4-Me_2_-Gal*p*	1,2,6-	26.1	27.0	99,113,129,159,189,233
2,3,4,6-Me_4_-Man*p*	T-	28.1	28.8	101,117,129,145,161,205
3,4,6-Me_3_-Man*p*	1,2-	10.4	4.6	87,101,129,145,161,189

In WGFP-A-a, Glc is mostly present as 1,6-Glc with additional contributions from 1,3-Glc, 1,3,6-Glc, 1,4-Glc, and T-Glc. The branching degree was found to be 25.2%. In addition to Glc, Man was present in the form of T-Man. We propose that WGFP-A-a contains a branched 1,6-glucan linked with T-Man*p* and T-Glc*p* at the O-3 position, whereas a small amount of 1,3-linked Glc may be present in either the side chains or main chain. In AGFP-A-b and AGFP-A-c, there are differences in the amount of Glc, which can exceed 90%. Glc mostly exists in the form of 1,6-linked. Additionally, Glc is also present in 1,3-, T-, and 1,4-linkages with degrees of branching of 42.3% and 37.6%. We suggest that AGFP-A-b and AGFP-A-c constitute a branched 1,6-glucan containing T-Glc*p* as side chains, whereas a small amount of 1,3-linked Glc is present in side chains or in the main chain (Table 3).

**TABLE 3:** Types and ratios of glycosidic bonds of WGFP-A-a, AGFP-A-b, and AGFP-A-c.

Methylated sugar	Linkage	Molar ratio (%)			Mass fragment (m/z)
		WGFP-A-a	AGFP-A-b	AGFP-A-c	
2,3,4,6-Me_4_-Glc*p*	T-	8.2	17.9	18.3	101,117,129,145,161,205
2,4,6-Me_3_-Glc*p*	1,3-	19.5	23.3	23.3	87,101,117,129,161,233
2,3,6-Me_3_-Glc*p*	1,4-	8.4	7.5	4.9	58,101,117,129,161,233
2,3,4-Me_3_-Glc*p*	1,6-	42.1	28.3	33.4	87,101,117,129,161,233
2,4-Me_2_-Glc*p*	1,3,6-	14.2	20.8	20.1	87,117,129,139,189,233
2,3,4,6-Me_4_-Man*p*	T-	7.6	2.2	—	101,117,129,145,161,205

#### 3.5.2 NMR spectra

**FIGURE 3:**
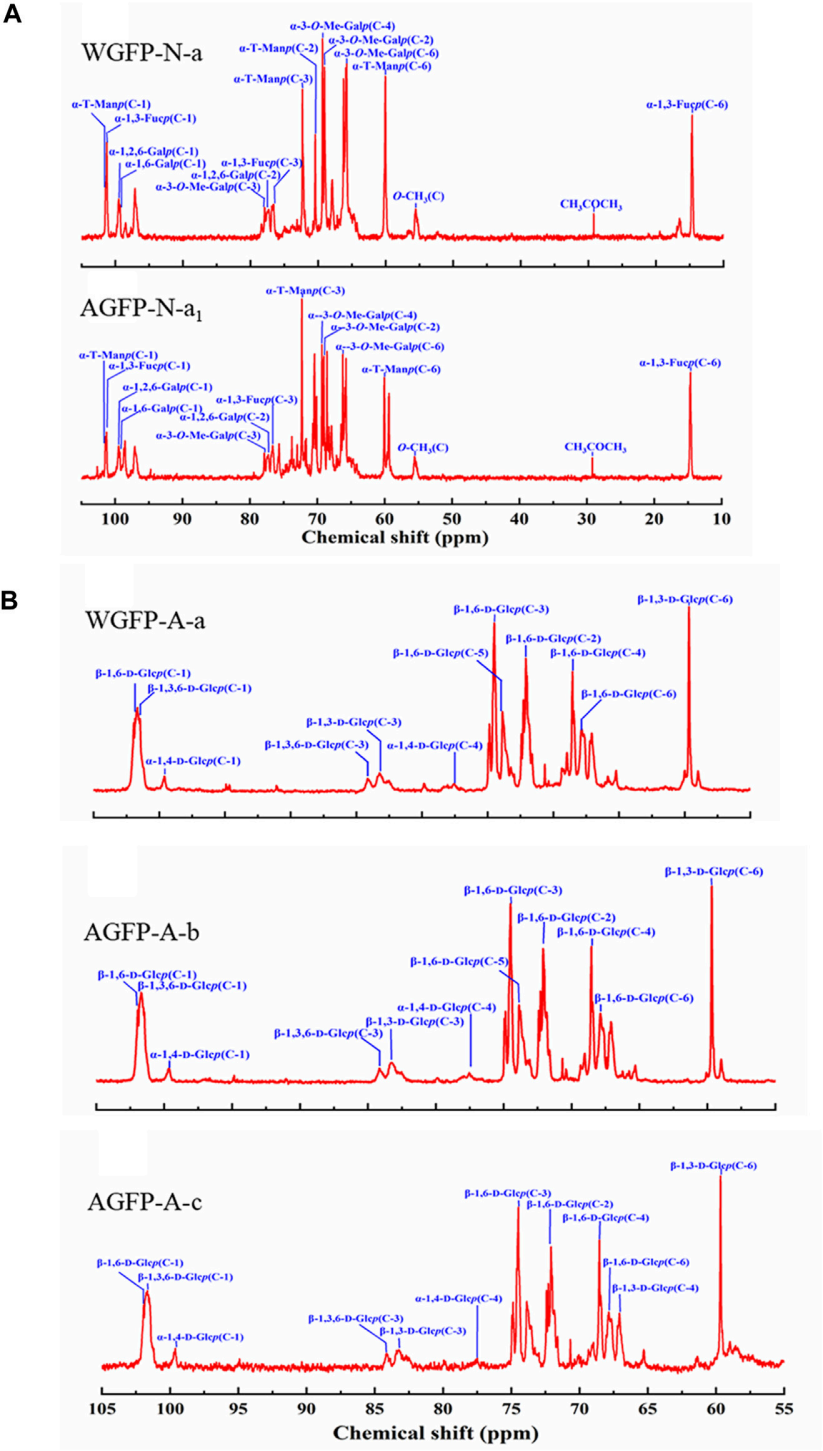
^13^C-NMR spectra of **(A)** WGFP-N-a and AGFP-N-a_1_; **(B)** WGFP-A-a, AGFP-A-b, and AGFP-A-c.

^13^C-NMR spectra of WGFP-N-a and AGFP-N-a_1_ are shown in Figure 3A, with chemical shift assignments presented in Table 4. Anomeric carbon signals of α-T-Man*p* in WGFP-N-a and AGFP-N-a_1_ were observed at 101.41 ppm and 101.42 ppm, while those of α-1,3-L-Fuc*p* were detected at 101.27 and 101.42 ppm, respectively. In addition, anomeric carbon signals of α-1,6-Me-D-Gal*p*, α-1,6-D-Gal*p*, and α-1,2,6-D-Gal*p* of WGFP-N-a appeared at 98.48, 99.30, and 99.51 ppm, while those of AGFP-N-a_1_ were found at 98.93, 99.29, and 99.49 ppm. In WGFP-N-a and AGFP-N-a_1_, signals from O-CH_3_ were observed at 55.58 ppm, whereas the presence of two signals near 77.90 ppm suggested that O-CH_3_ was connected to the O-3 position of Gal. The existence of Me-Gal was confirmed, which is consistent with the monosaccharide composition detected by HPLC. Taken together with methylation results, we propose that WGFP-N-a and AGFP-N-a_1_ primarily consist of α-1,6-D-Me-Gal*p* as their main chains with a small amount of α-1,6-D-Gal*p* branched at the O-2 position. α-1,3-L-Fuc*p* and α-T-Man*p* are likely present at the side chains. Therefore, these polysaccharides can be called fucomannogalactan.

**TABLE 4:** ^13^C-NMR spectral assignments of WGFP-N-a, AGFP-N-a_1_, WGFP-A-a, AGFP-A-b, and AGFP-A-c.

Fraction	Linkage type	C-1	C-2	C-3	C-4	C-5	C-6	O-CH_3_
WGFP-N-a	α-1,6-D-Gal*p*	99.30	69.05	68.55	69.38	67.96	65.90	—
	α-1,2,6-D-Gal*p*	99.51	77.32	68.43	67.90	67.83	65.81	—
	α-1,6-D-Me-Gal*p*	98.48	69.05	77.91	69.36	67.90	65.76	55.57
	α-T-Man*p*	101.41	70.44	72.32	69.30	74.71	60.07	—
	α-1,3-L-Fuc*p*	101.27	70.69	76.65	73.10	67.17	14.76	—
AGFP-N-a_1_	α-1,6-D-Gal*p*	99.29	69.04	68.55	69.31	67.96	66.26	—
	α-1,2,6-D-Gal*p*	99.49	77.27	68.42	67.90	67.71	66.07	—
	α-1,6-D-Me-Gal*p*	98.93	69.04	77.88	69.31	67.92	65.90	55.59
	α-T-Man*p*	101.42	70.42	72.30	69.27	74.71	60.09	—
	α-1,3-L-Fuc*p*	101.29	70.68	76.62	73.00	67.16	14.73	—
WGFP-A-a	β-1,6-D-Glc*p*	101.85	72.09	74.49	68.54	73.87	67.70	—
	β-1,3,6-D-Glc*p*	101.47	72.31	84.13	68.45	74.86	67.84	—
	β-1,3-D-Glc*p*	101.68	71.62	83.22	67.07	74.49	59.67	—
	α-1,4-D-Glc*p*	99.61	71.63	73.39	77.54	71.38	60.48	—
AGFP-A-b	β-1,6-D-Glc*p*	101.94	72.00	74.49	68.55	73.87	67.70	—
	β-1,3,6-D-Glc*p*	101.69	72.28	84.25	68.46	74.87	67.84	—
	β-1,3-D-Glc*p*	101.78	71.62	83.24	67.10	74.49	59.68	—
	α-1,4-D-Glc*p*	99.67	71.64	73.37	77.53	71.36	60.32	—
AGFP-A-c	β-1,6-D-Glc*p*	101.94	72.09	74.51	68.54	73.87	67.70	—
	β-1,3,6-D-Glc*p*	101.52	72.28	84.17	68.46	74.87	67.88	—
	β-1,3-D-Glc*p*	101.67	71.83	83.23	67.10	74.49	59.67	—
	α-1,4-D-Glc*p*	99.56	71.63	73.36	77.54	71.38	60.48	—

^13^C-NMR spectra of WGFP-A-a, AGFP-A-b, and AGFP-A-c are shown in Figure 3B, and their chemical shifts are provided in Table 4. In WGFP-A-a, AGFP-A-b, and AGFP-A-c, anomeric carbon signals from β-1,6-D-Glc*p* are observed at 101.85, 101.94, and 101.94 ppm, respectively. Anomeric carbon signals from β-1,3,6-D-Glc*p* appeared at 101.47 ppm, 101.69, and 101.52 ppm, whereas those from α-1,4-D-Glc*p* are found at 99.61, 99.67, and 99.56 ppm, respectively. The structures of these three polysaccharides are similar and are consistent with our methylation results. We concluded that WGFP-A-a, AGFP-A-b, and AGFP-A-c are composed of β-1,6-D-Glc*p* as the main chain that is substituted at O-3 position. β-1,3-D-Glc*p* may exist in side chains or in the main chain.

^1^H-NMR also showed the same results similar to the ^13^C-NMR spectra, which further confirmed the structure of polysaccharides. More detailed analysis of ^1^H-NMR spectra of WGFP-N-a, AGFP-N-a_1_, WGFP-A-a, AGFP-A-b, and AGFP-A-c is shown in the Supplementary Material (Supplementary Results; Supplementary Figure S9; Supplementary Table S2).

**FIGURE 4:**
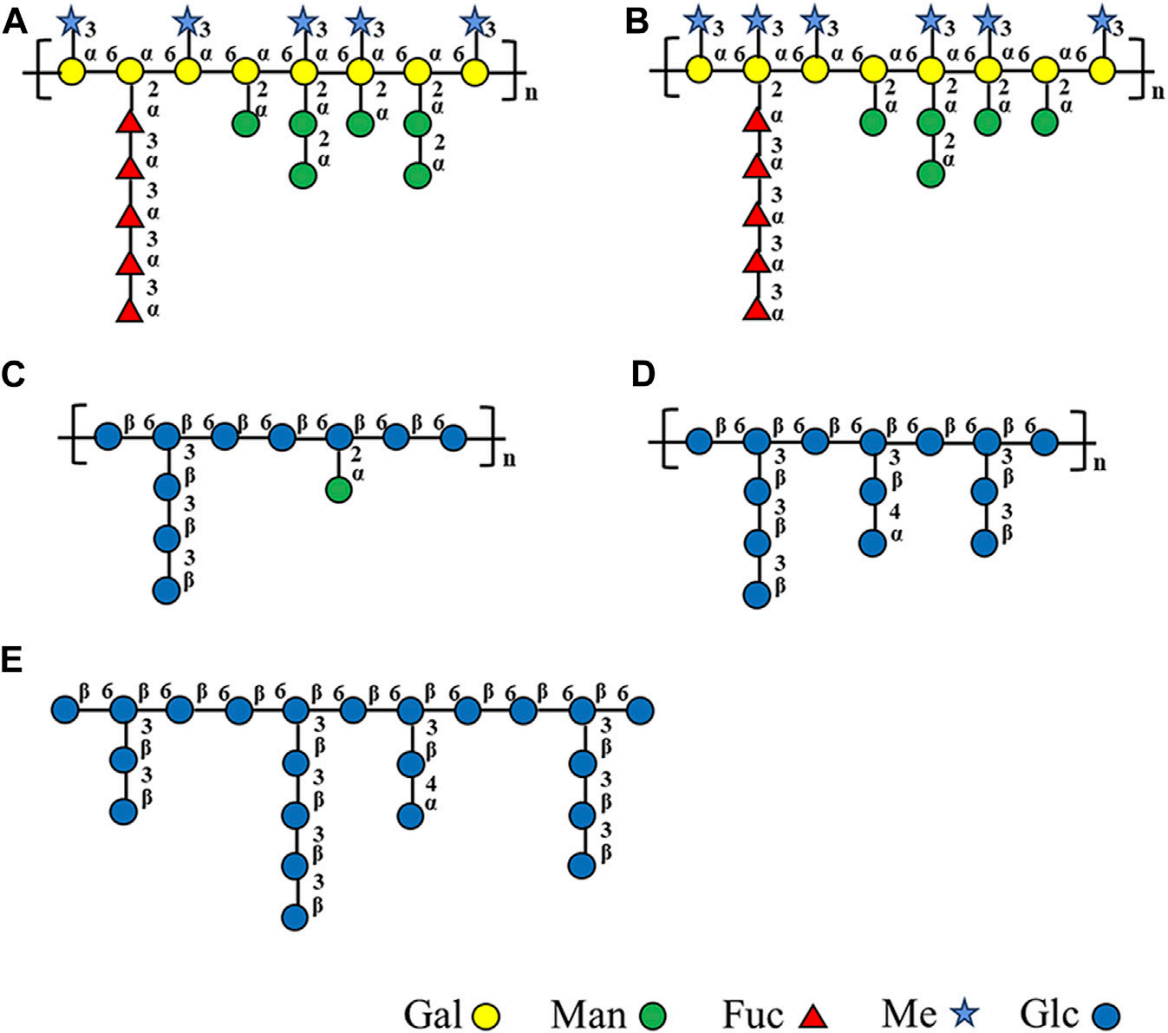
Structural models of **(A)** WGFP-N-a, **(B)** AGFP-N-a_1_, **(C)** WGFP-A-a, **(D)** AGFP-A-b, and **(E)** AGFP-A-c.

Based on monosaccharide composition, methylation, FT-IR, and NMR, Figure 4 illustrates structural models of the homogeneous fractions of these five polysaccharides.

## 4 Discussion

*Grifola frondosa* is a valuable, medicinal fungus, with a rich nutritional value. Polysaccharides are one of the most bioactive substances derived from *G. frondosa* (He et al., 2017; Wu et al., 2021), yet this fungus is limited in nature and difficult to cultivate. This limits the amount of fruiting bodies that can be obtained to process large quantities of its polysaccharides. However, the use of submerged cultures of this fungus offers a promising alternative as it is rapid, cost-effective, easy to control, and free from heavy metal contamination. Here, *G. frondosa* mycelium-derived polysaccharides were extracted by alkylation, acidification, or simply by using water. Iino et al. (1985) were the first to extract Grifolan from *G. frondosa* mycelium under cold alkaline conditions. Adachi et al. (1994) extracted β-1,3-D-glucan from *G. frondosa* mycelium using 0.5% citrate buffer. Zhao et al. (2016) extracted polysaccharide GRP1 using hot water. It has been reported that different extraction methods resulted in different Mw compositions and structures of polysaccharides (Ren et al., 2023). However, there is no report on the extraction of polysaccharides from mycelium residues by alkaline solution after hot water extraction of polysaccharides from *G.* *frondosa* mycelium. In the present study, we used this approach for the first time to thoroughly extract *G. frondosa* mycelium-derived polysaccharides.

Previous reports showed that *G. frondosa* mycelium-derived polysaccharides are mainly composed of α-1,3-Glc*p*, α-1,6-Glc*p*, and α-1,4-Gal*p* as the main chains. Similar structured polysaccharides (termed WGFP-A-a, AGFP-A-b, and AGFP-A-c) were obtained in this study. These are composed of β-1,6-D-Glc*p* and β-1,3-D-Glc*p*, with T-D-Glc*p* likely present in the side chains to form β-1,6-glucan. The molecular weights of these three polysaccharides ranged from 4.1 kDa to 19.9 kDa, similar to GFPS1b with a molecular weight of 21 kDa extracted by Cui et al. (2007) and smaller than GRP1 (40.5 kDa) extracted by Zhao et al. (2016).

Zhang et al. (2023) extracted an acidic polysaccharide GFP-A from the fruiting bodies of *G. frondosa*. The molecular weight of GFP-A was reported to be ∼1,100 kDa, making it much larger than the mycelium-derived polysaccharide identified in this study. GFP-A is mainly composed of Glc with α-type glycosidic linkages, whereas WGFP-A-a, AGFP-A-b, and AGFP-A-c are mainly composed of β-Glc*p*. Glucan GFPA (∼5,570 kDa) was extracted from the fruiting bodies of *G. frondosa* (Li et al., 2022) and has an α-1,4-D-Glc*p* backbone with β-1,4,6-D-Glc*p* and T-β-Glc*p* as side chains. The mycelium glucans found in this study have lower molecular weights (4.1 kDa to ∼19.9 kDa) and are composed of a β-1,6-D-Glc*p* backbone with branched side chains of β-1,3-D-Glc*p* and T-D-Glc*p* residues at O-3 of the Glc*p* residues.

It is noteworthy that two novel polysaccharides WGFP-N-a and AGFP-N-a_1_ are reported here. These are primarily composed of α-1,6-D-Me-Gal*p* and α-1,6-D-Gal*p* as main chains. WGFP-N-a and AGFP-N-a_1_ are fucomannogalactan because they contain Fuc and Man as side chains. This structure has been reported in *G. frondosa* fruiting bodies but was not yet found in *G. frondosa* mycelia (Wang et al., 2014). Our present research provides the basis for extracting fucomannogalactan and β-1,6-glucans from *G. frondosa* mycelium.

## 5 Conclusion

In this study, we have isolated polysaccharides from *G. frondosa* mycelium and analyzed five homogeneous fractions that are divided into two types of structures: fucomannogalactan and β-1,6-glucans. WGFP-N-a and AGFP-N-a_1_ mainly consist of α-1,6-D-Me-Gal*p*, with a small amount of α-1,6-D-Gal*p* as the main chain and with α-1,3-L-Fuc*p*, α-1,2-D-Man*p*, and α-T-D-Man*p* as side chains branched at the O-2 position of Me-Gal and Gal, to form fucomannogalactan. WGFP-A-a, AGFP-A-b, and AGFP-A-c are mainly composed of β-1,6-D-Glc*p* branched at O-3 of Glc. β-1,3-D-Glc*p* and T-D-Glc*p* may exist as side chains to form β-1,6-glucan. To the best of our knowledge, this is the first study that has characterized polysaccharides derived from *G. frondosa* mycelium.

## Data Availability

The original contributions presented in the study are included in the article/Supplementary Material; further inquiries can be directed to the corresponding author.
