# Supplementary material for: Preparation and structural analysis of fucomannogalactan and β-1,6-glucan from Grifola frondosa mycelium
Source: Front Chem. 2023 Aug 7;11:1227288. doi: 10.3389/fchem.2023.1227288 (PMC10441114; doi:10.3389/fchem.2023.1227288)
Supplement: Supplementary file 1 [file DataSheet1.docx]

Supplementary Material

**Preparation and structural analysis of fucomannagalactan and β-1,6-glucan from *Grifola frondosa* mycelium**

**Jie Geng^1^, Guining Wang^1^, Jiao Guo^1^, Xiao Han^1^, Yunhe Qu^2^, Yifa Zhou^1^, Guihua Tai^1^, Lin Sun^1^, Hairong, Cheng^1^***

*** Correspondence:** Hairong, Cheng：chenghr893@nenu.edu.cn

# Supplementary Results

The ^1^H-NMR spectra of WGFP-N-a and AGFP-N-a_1_ were shown in Supplementary Figure 9A, and the chemical shift assignments were shown in Supplementary Table 2. Signals at 4.96 ppm and 5.09/5.10 ppm were assigned to H-1 resonances of α-1,6-D-Gal*p* and α-1,2,6-D-Gal*p*, respectively. Signals at 5.05 ppm were assigned to H-1 resonances of α-1,3-L-Fuc*p*, whereas H-6 of α-1,3-L-Fuc*p* was observed at 1.17 ppm.

The ^1^H-NMR spectra of WGFP-A-a, AGFP-A-b and AGFP-A-c were shown in Supplementary Figure 9B, and the chemical shift assignments were shown in Supplementary Table 2. Signals at 4.48 ppm and 4.47 ppm were assigned to H-1 resonances of β-1,6-D-Glc*p* and β-1,3,6-D-Glc*p*, respectively, whereas the ^1^H peak at 4.70 ppm was assigned to the anomeric proton resonance of β-1,3-D-Glc*p* or β-T-D-Glc*p*.

# Supplementary Figures and Tables

## Supplementary Figures

**
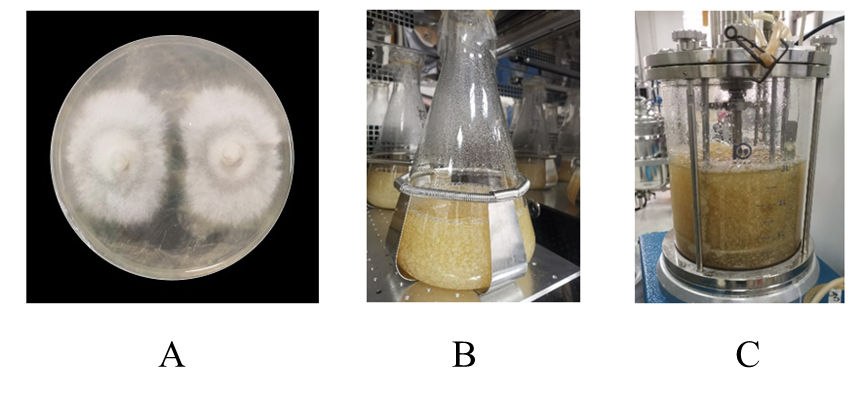
**

**Supplementary Figure 1.** The morphology of *Grifola frondosa* mycelium （A）solid activation culture; (B) liquid seed culture; (C) fermenter culture.


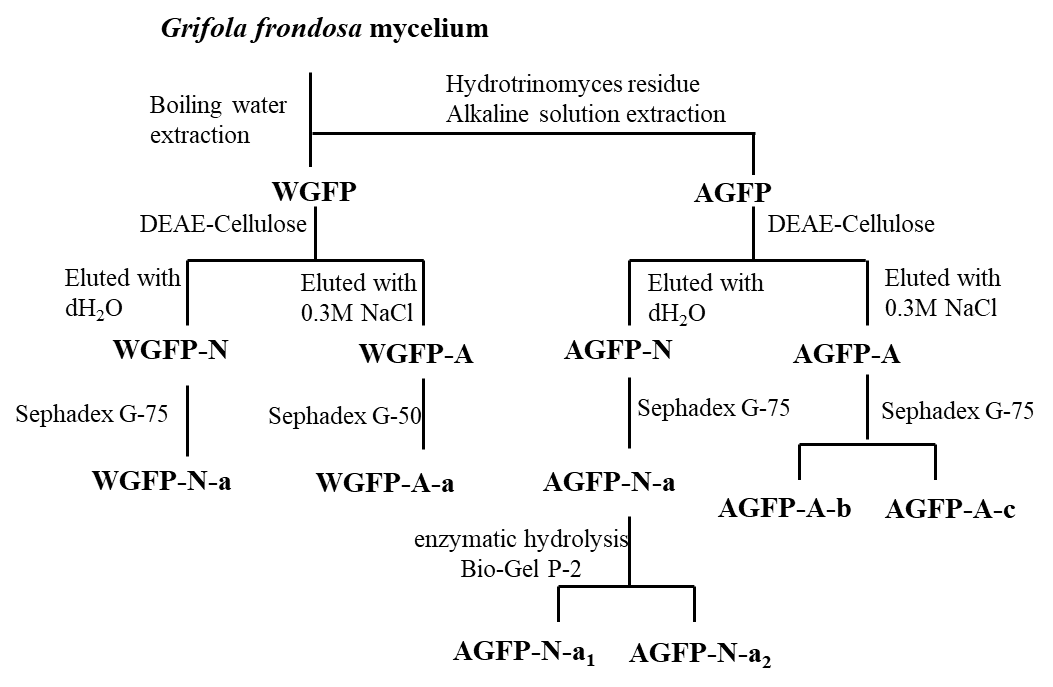


**Supplementary Figure 2.** Schematic of polysaccharide extraction and separation and purification.


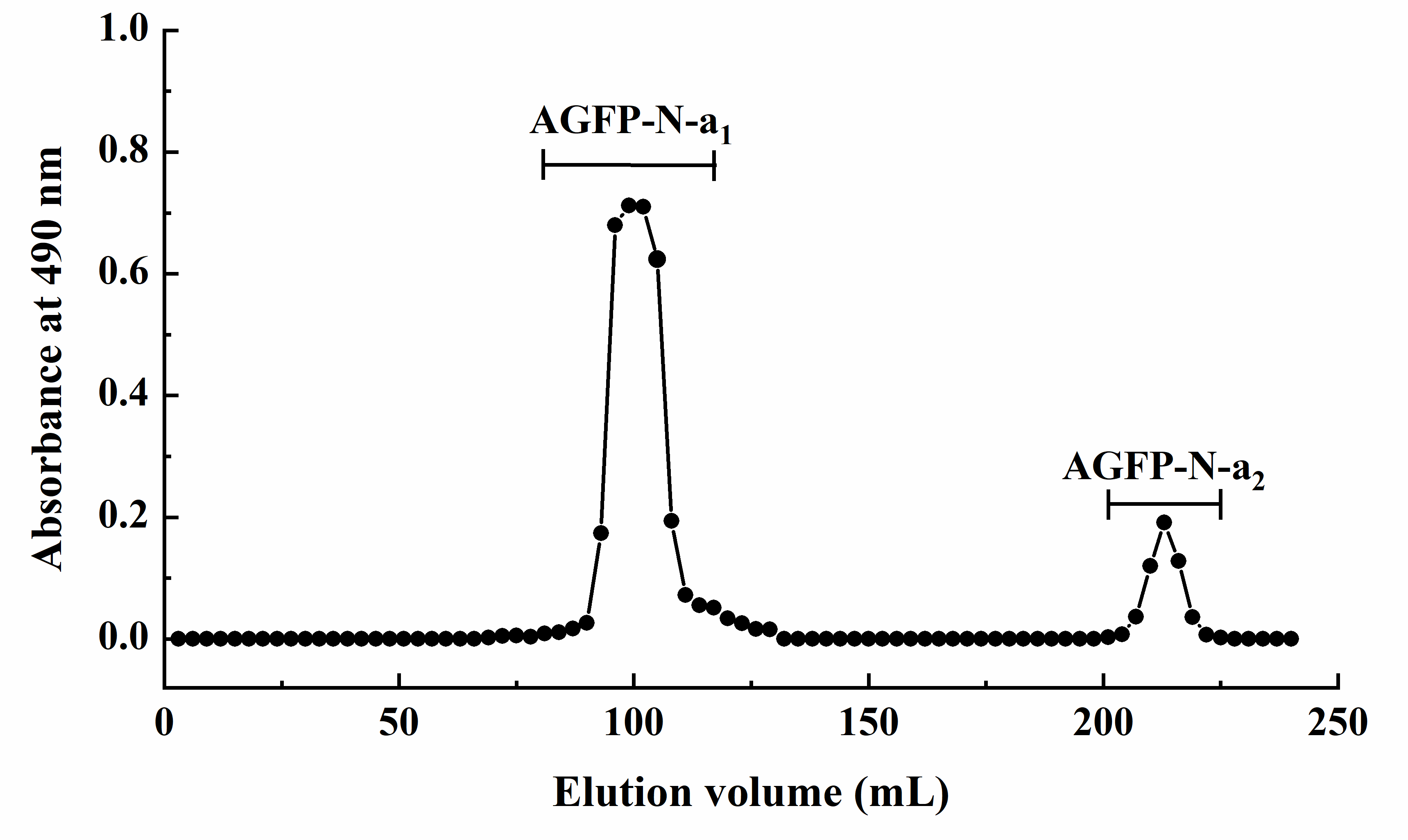


**Supplementary Figure 3.** Elution profiles of AGFP-N-a on Bio-Gel P-2 column, eluted by ddH_2_O (-●-, total sugar) .


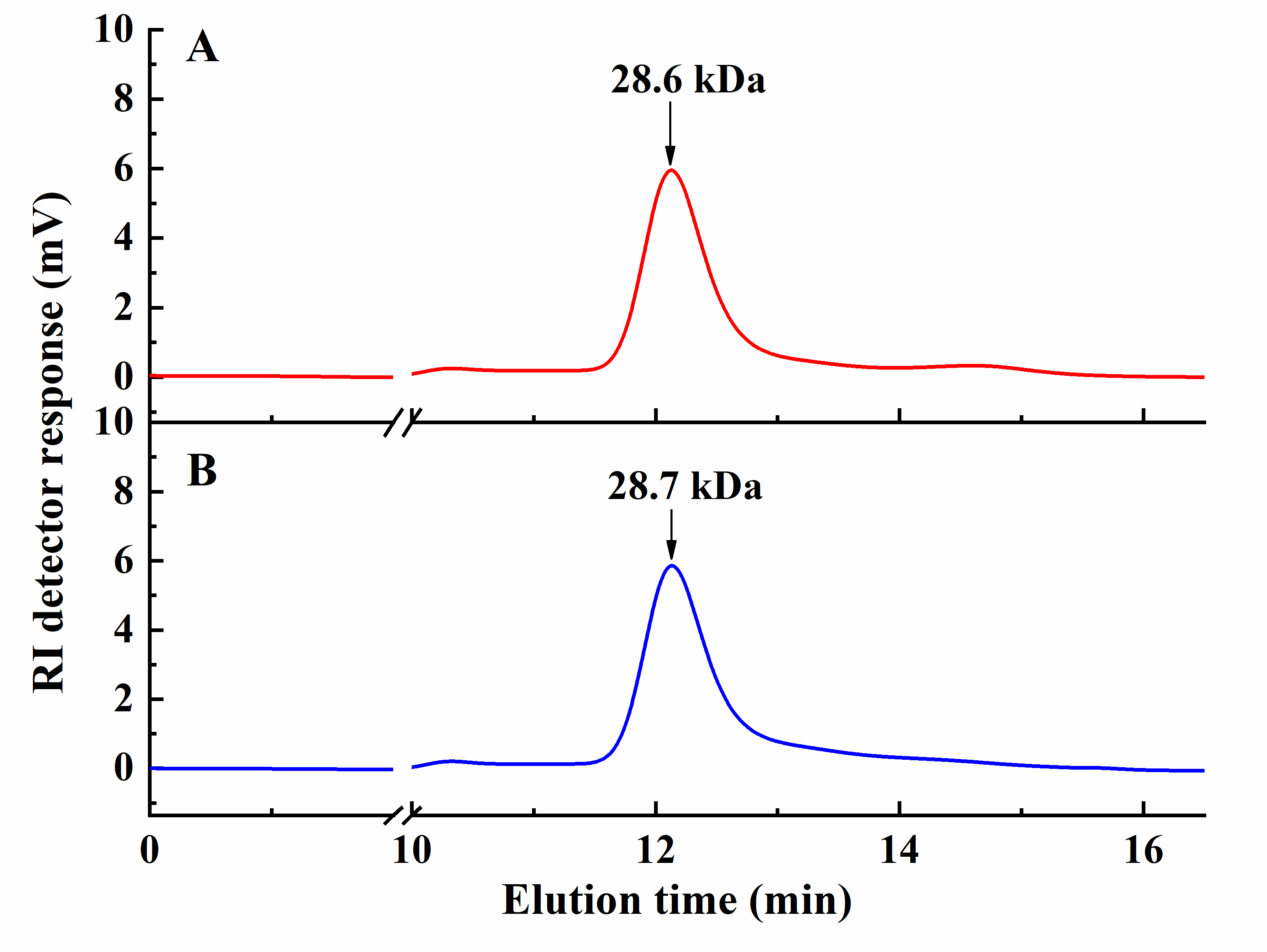


**Supplementary Figure 4.** HPGPC profiles of molecular weight distribution of (A) AGFP-N-a and (B) AGFP-N-a_1_.


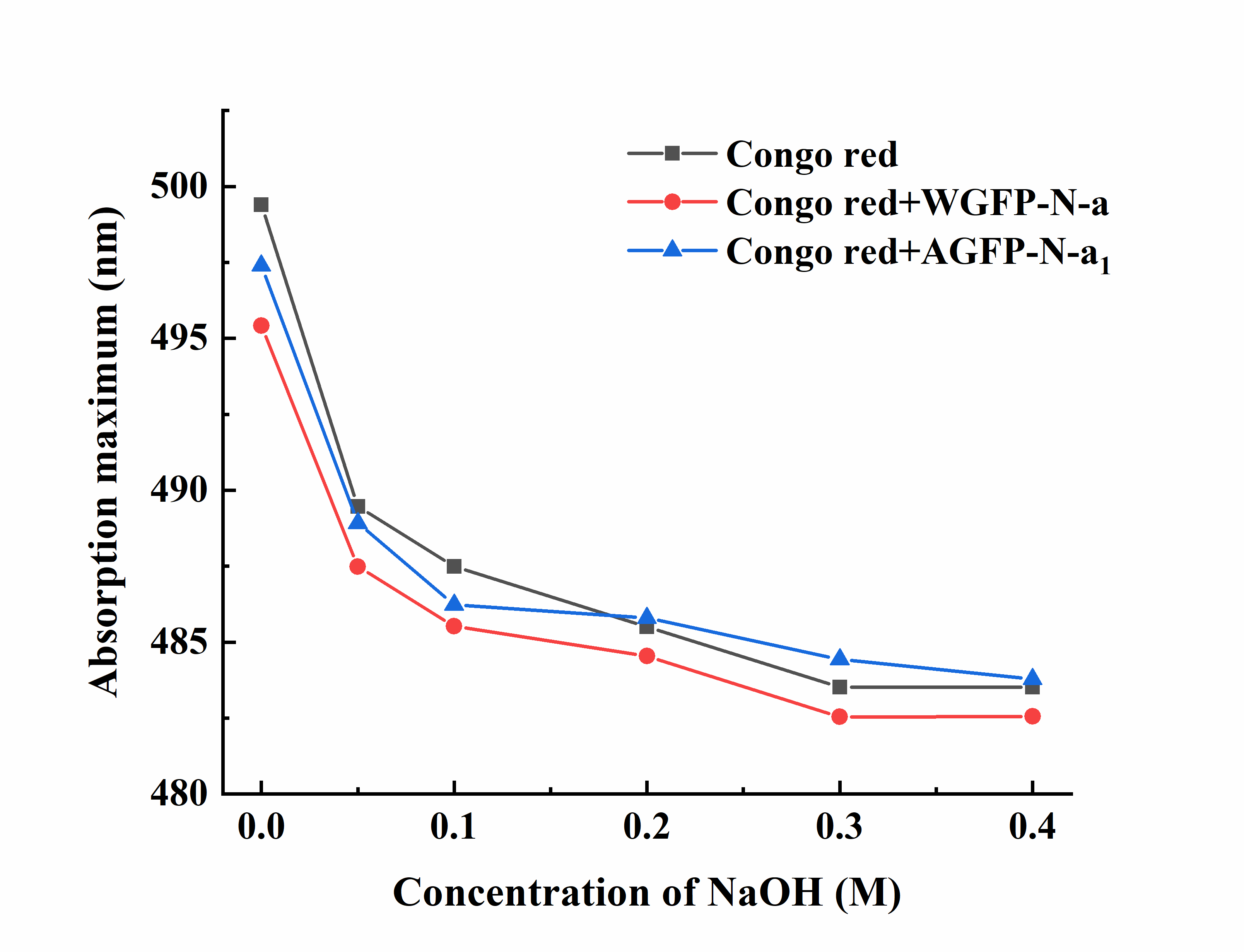


**Supplementary Figure 5.** Trihelix structure analysis diagram of WGFP-N-a and AGFP-N-a_1_.


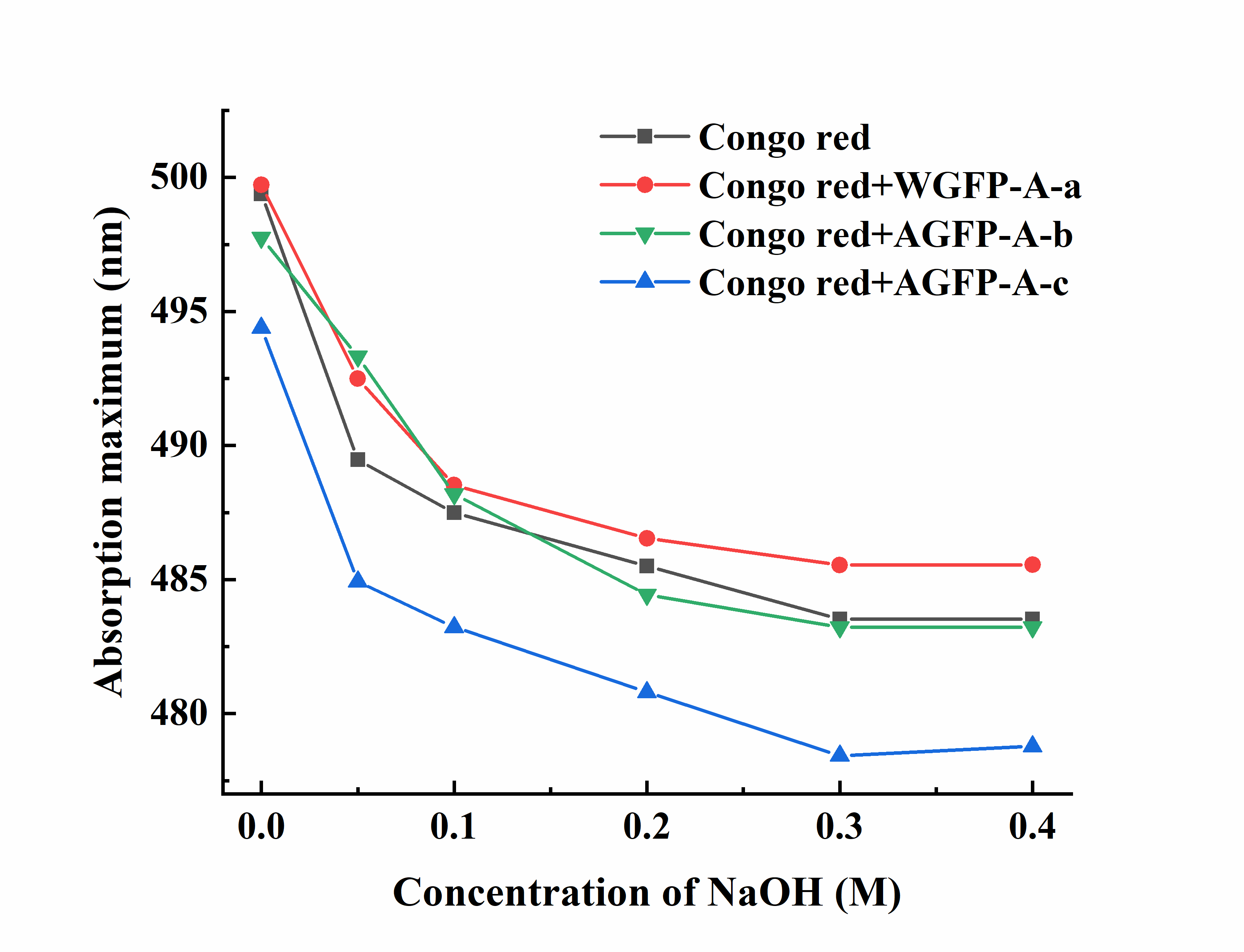


**Supplementary Figure 6.** Trihelix structure analysis diagram of WGFP-A-a, AGFP-A-b and AGFP-A-c.


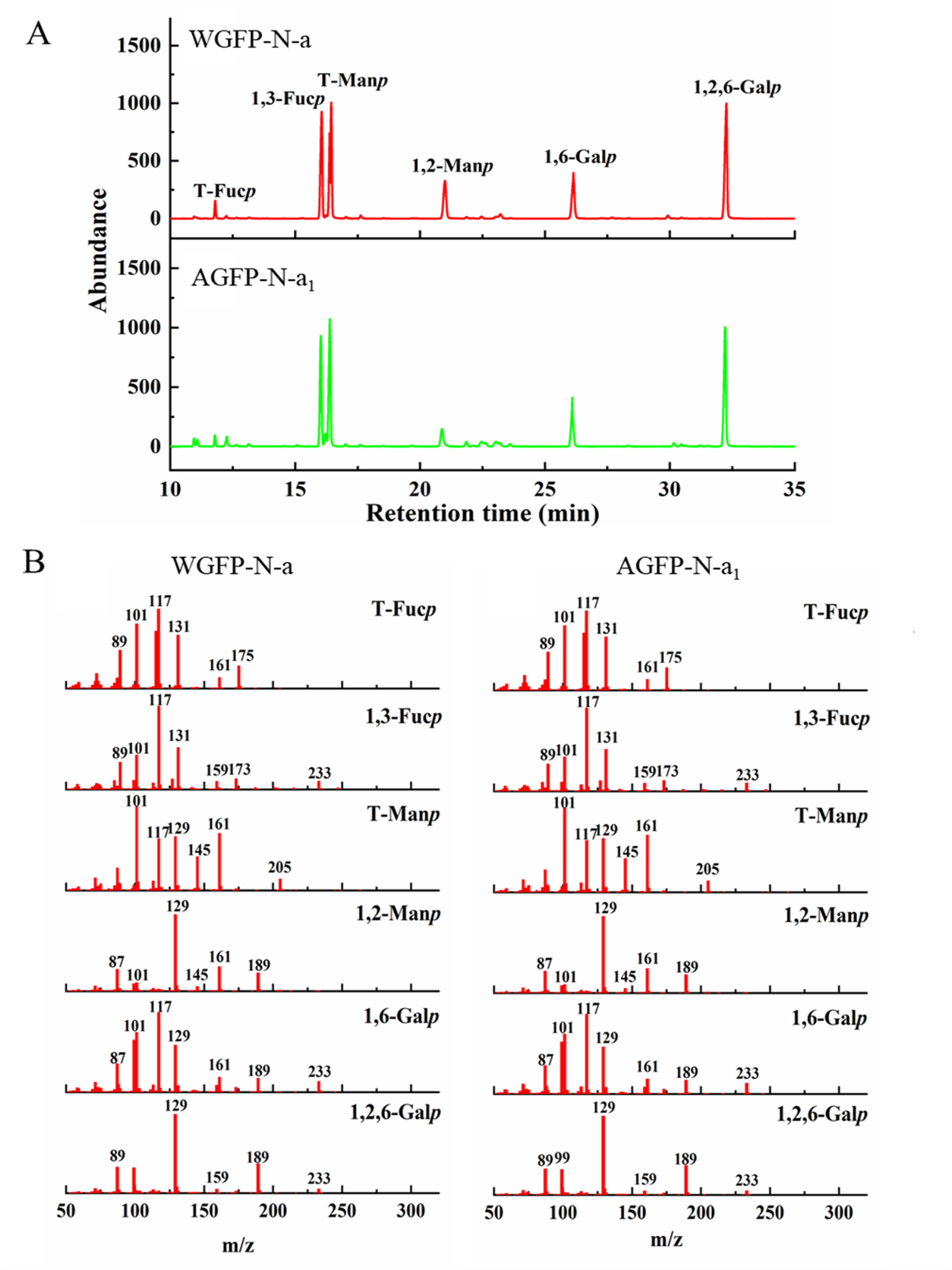


**Supplementary Figure 7.** The GC-MS total ion chromatogram (A) and mass spectrum of partially methylated alditol acetates derivatives (B) from of WGFP-N-a and AGFP-N-a_1_.


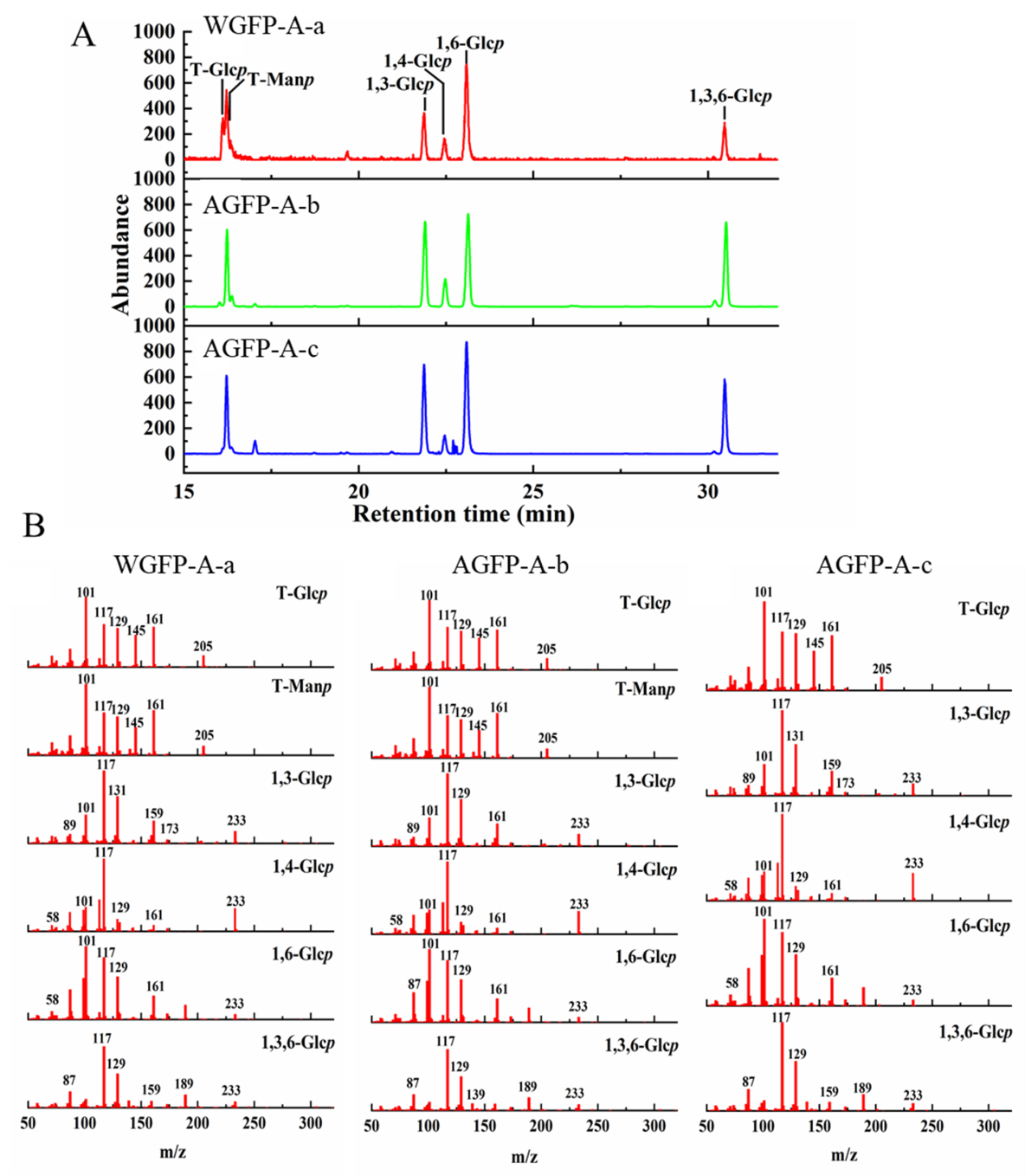


**Supplementary Figure 8.** The GC-MS total ion chromatogram (A) and mass spectrum of partially methylated alditol acetates derivatives (B) from of WGFP-A-a, AGFP-A-b and AGFP-A-c.


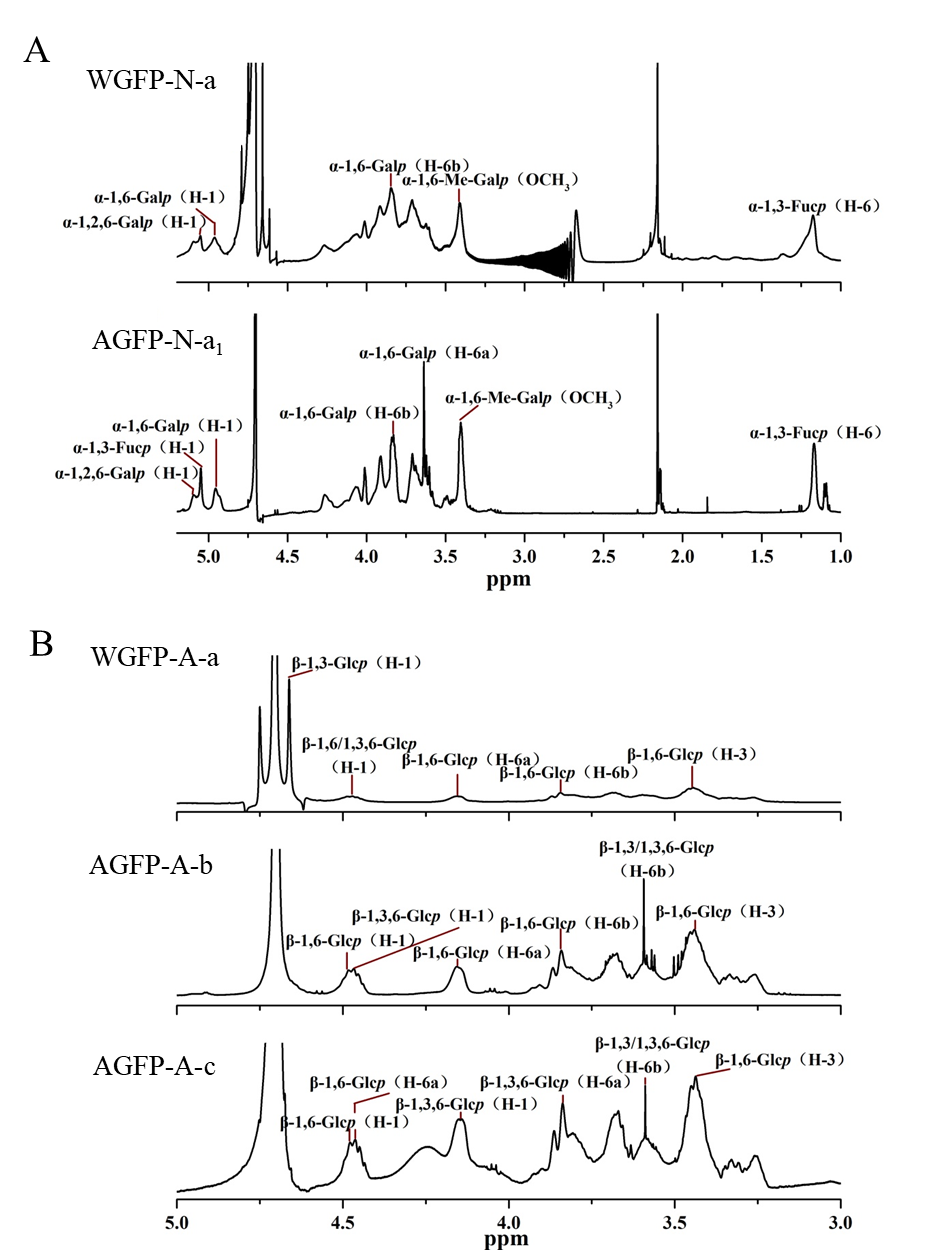


**Supplementary Figure 9.** The ^1^H-NMR spectrum of (A) WGFP-N-a and AGFP-N-a_1_, (B) WGFP-A-a, AGFP-A-b and AGFP-A-c.

## Supplementary Table

**Supplementary Table 1.** Composition of total polysaccharides in *Grifola frondosa* mycelium extracted by water and alkali

| Composition | Total sugar (%) | Uronic acid (%) | Protein (%) | | Ash (%) |
| --- | --- | --- | --- | --- | --- |
|  |  |  | Before deproteinization | After deproteinization |  |
| WGFP | 42.3 | 3.5 | 18.9 | 3.2 | 33.0 |
| AGFP | 49.8 | 3.6 | 28.8 | 4.1 | 5.7 |

**Supplementary Table 2.** ^1^H-NMR spectral assignments of WGFP-N-a, AGFP-N-a_1_, WGFP-A-a, AGFP-A-b and AGFP-A-c

| Fractions | Linkage type | H-1 | H-2 | H-3 | H-4 | H-5 | H-6 | O-CH_3_ |
| --- | --- | --- | --- | --- | --- | --- | --- | --- |
| WGFP-N-a | α-1,6-D-Gal*p* | 4.96 | 3.84 | 4.01 | 4.06 | 4.12 | 3.84, 3.62 | -- |
|  | α-1,2,6-D-Gal*p* | 5.09 | 3.92 | 4.01 | 3.92 | 3.72 | 3.84, 3.61 | -- |
|  | α-1,6-D-Me-Gal*p* | 4.96 | 3.84 | 3.50 | 4.27 | 4.12 | 3.84, 3.62 | 3.41 |
|  | α-1,3-L-Fuc*p* | 5.05 | -- | -- | -- | -- | 1.17 | -- |
| AGFP-N-a_1_ | α-1,6-D-Gal*p* | 4.96 | 3.81 | 4.01 | 4.06 | 4.12 | 3.84, 3.63 | -- |
|  | α-1,2,6-D-Gal*p* | 5.10 | 3.91 | 4.01 | 3.91 | 3.81 | 3.84, 3.62 | -- |
|  | α-1,6-D-Me-Gal*p* | 4.96 | 3.82 | 3.50 | 4.27 | 4.12 | 3.83, 3.60 | 3.40 |
|  | α-1,3-L-Fuc*p* | 5.05 | -- | -- | 3.71 | 4.12 | 1.17 | -- |
| WGFP-A-a | β-1,6-D-Glc*p* | 4.48 | 3.26 | 3.46 | 3.44 | 3.60 | 4.16, 3.81 | -- |
|  | β-1,3,6-D-Glc*p* | 4.47 | 3.46 | 3.68 | 3.34 | 3.84 | 3.87, 3.68 | -- |
|  | β-1,3-D-Glc*p* | 4.66 | 3.60 | 3.68 | 3.44 | 3.76 | 3.87, 3.68 | -- |
|  | β-T-D-Glc*p* | 4.66 | 3.34 | 3.26 | 3.60 | 3.84 | 3.87, 3.68 | -- |
| AGFP-A-b | β-1,6-D-Glc*p* | 4.48 | 3.26 | 3.45 | 3.44 | 3.57 | 4.16, 3.81 | -- |
|  | β-1,3,6-D-Glc*p* | 4.47 | 3.48 | 3.69 | 3.44 | 3.84 | 3.91, 3.70 | -- |
|  | β-1,3-D-Glc*p* | 4.70 | 3.59 | 3.63 | 3.49 | 3.76 | 3.91, 3.69 | -- |
|  | β-T-D-Glc*p* | 4.70 | 3.35 | 3.26 | 3.63 | 3.84 | 3.91, 3.69 | -- |
| AGFP-A-c | β-1,6-D-Glc*p* | 4.48 | 3.26 | 3.45 | 3.44 | 3.56 | 4.14, 3.81 | -- |
|  | β-1,3,6-D-Glc*p* | 4.46 | 3.50 | 3.66 | 3.44 | 3.84 | 3.86, 3.67 | -- |
|  | β-1,3-D-Glc*p* | 4.67 | 3.56 | 3.63 | 3.50 | 3.75 | 3.90, 3.67 | -- |
|  | β-T-D-Glc*p* | 4.67 | 3.35 | 3.26 | 3.63 | 3.84 | 3.90, 3.67 | -- |
